# The effect of work area on work alienation among China’s grassroots judicial administrators

**DOI:** 10.1038/s41598-022-23526-w

**Published:** 2022-11-05

**Authors:** Nian Liu, Meiling Zhang, Boya Feng

**Affiliations:** 1 Department of Sociology, Guangzhou University, Guangzhou, 510006 China; 2 Social Construction Committee of Guangdong Provincial People’s Congress, Guangzhou, 510062 China; 3 Department of Social Work, Tunghai University, Taichung, 407224 Taiwan; 4 School of Law, Guangdong University of Technology, Guangzhou, 510520 China

**Keywords:** Human behaviour, Health occupations

## Abstract

Work alienation refers to mental self-separation from work, and it is an integral reflection of workers and their work. Few studies have explored the association between work area and work alienation among grassroots judicial administrators. A stratified sampling method was used to collect data from 288 grassroots judicial administrators in Guangzhou to measure the overall status quo and work alienation in this group. This study found that the current grassroots judicial administrative team has a reasonable structure, high professional quality, and rich grassroots work experience, but a high level of work intensity (Mean = .667) and a lack of autonomy (Mean = .757) are prominent. Work area (unstandardized regression coefficient, B = .917) is significantly related to work alienation when controlling for sociodemographic and work characteristic variables: the closer the work area is to the city centre areas, the higher the level of work alienation. In addition, education level also has a significant effect on work alienation: the lower the individual education level is, the stronger the work alienation. The discussion focuses on the knowledge needs in grassroots judicial administrative work and the importance of the external working environment, and further research implications are proposed.

## Introduction

Work alienation refers to a mental state when the working environment does not meet the work expectations and needs of employees, which leads to the isolation of employees from the work they are engaged in^1^. It also refers to workers’ loss of purpose at work^2^. As an important academic concept, work alienation has long been used in research on many topics, such as workers’ occupational psychology^3,4^, occupational mobility^5,6^ and interaction with their organizational environment^7,8^. Work alienation is the overall reflection of a worker’s relationship with the people, organization, society, and environment surrounding his or her work. It is affected by the individual characteristics of employees^9^, psychological controls, work characteristics^1^, leadership styles^10^, working environments^11^ and many other factors. In recent decades, work alienation has been a focal topic in Chinese sociology and psychology research^12–14^.

China is in a period of deepening social transformation. With the acceleration of social changes, the instability of the relation between workers and their jobs has increased. After the 1990s, the social function attributes attached to work, such as housing, children’s education, and medical care, were stripped away, and work returned to the pure professional relationship itself; in recent years, “996” (workers go to work at 9 a.m. and return from work at 9 p.m., 6 days a week) and other work cultures that represent high-intensity work patterns have prevailed. The distance between workers and their work is ever increasing, and the problem of individual alienation caused by work has been widely commented on in academic and practical circles.

Chinese grassroots judicial administrators are stationed in community judicial offices. The judicial office is the most basic organization of the national judicial administrative authority and is the community-facing agency at the town level (subdistrict) of the municipal (district, county) judicial bureau responsible for the implementation of various grassroots judicial administrative tasks. Grassroots judicial administrators have the closest contact with the community and are responsible for resolving community conflicts, advancing the rule of law in the community, supervising offenders in community corrections, and maintaining the harmony and stability of local society. At present, there are 40,513 judicial offices in China and 128,000 grassroots judicial administrators, covering all towns (subdistricts) nationwide^15^. Domestic and foreign research has rarely paid attention to this unique and sizeable occupational group. In the judicial system, consisting of public security—procuratorate—court—judicial administration, judicial administrative work is characterized by serviceability, triviality and complexity relative to the former three; comparatively speaking, as the stability of judicial administrative staff in the judicial system is the worst, a large number of grassroots judicial administrative workers apply for post transfer and job transfer^16^.

Work alienation as a sensitive and effective indicator for explaining and predicting employee psychology and behaviour^6^ provides an in-depth research perspective for understanding the situation of this occupational group. China is a dual society of urban and rural areas; this classification is mainly based on spatial ranges formed by geographical regions and has significant influences on politics, economy, culture, and social psychology^17^. Theoretically, grassroots judicial workers face different community environment and community governance models in rural and urban areas. Thus, an empirical exploration has to be conducted to verify whether this kind of social structural difference gives rise to a distinct sense of work alienation among grassroots judicial administrators. This study takes grassroots judicial administrators in Guangzhou as the research object and takes work alienation as the entry point of the research. This study centres on the macro level of the community where the focal organization is located and probes the correlation between community environment, community governance model and work alienation. Its focus on the factors influencing work alienation encompasses the community environment outside the organization and work characteristics; moreover, countermeasures and suggestions for alleviating the sense of work alienation among grassroots judicial workers and stabilizing the judicial administrative team are put forward at a broader level in terms of the complexity of grassroots work and the community governance model. From the perspective of the external working environment, it aims to achieve the following research purposes: (1) understand the structuration of grassroots judicial administrators; (2) explore the status quo of their work alienation; (3) determine whether work area and other factors affect the level of work alienation of grassroots judicial administrators; and (4) interpret the results considering the characteristics of grassroots community governance and propose corresponding research countermeasures.

### Hypotheses

*Alienation theory and work area* The original meaning of the word “alienation” in English refers to a process in which people become unfamiliar with the world they live in, and it encompasses connotations of estrangement, detachment, separation, rule by alien forces, and domination by others^18^. In philosophy, it is generally translated as alienation, which refers to a state in which the opposing object is gradually separated from the subject in its activities, thereby forming an external alien force against the subject^19^. In sociology, it refers to the separation of the individual from the main aspects of his society. In economics, it refers to the various antagonistic relationships that people have in the economic field^20,21^. In the psychological sense, alienation was first used by Marx in his *Economic and Philosophic Manuscripts of 1844*, and Marx elaborated on the theory of alienation in his works. He noted that alienation is a complex concept that includes the two meanings of depersonalization and social estrangement^22^. Due to the influence of social changes and urban industrialization, the original harmony between people and their living environment has been lost, resulting in a sense of alienation in modern people.

The most striking thing about the theory of alienation is that it points out people’s social alienation from the perspective of human nature; that is, the loss of human nature leads to the generation of social separation. The traditional Marxist viewpoint emphasizes that labourers are objectively exploited and lack a sense of control and points out the subjective sense of powerlessness and self-separation of labourers. Therefore, the generation of alienation in labourers involves both subjective and objective factors. Subsequently, many scholars have refined Marx’s theory of alienation and expanded the concept. In the field of management psychology, researchers focus on the alienation between employees and work, which is called work alienation. Work alienation, as a type of alienation (others are cultural alienation, political alienation, etc.), reflects and is consistent with the theoretical origin of alienation theory and its core theoretical foundation, emphasizing the complex relationship between employees and their working environment^23^. In the field of organizational behaviour, researchers suggest that work alienation essentially reflects that the needs of employees are not satisfied by their work, and the source of alienation lies in the gap between the objective work situation and the employees’ values, ideals, and hobbies^1,24^.

Seeman^8^ conceived work alienation in five dimensions from the perspective of social psychology: labour alienation, powerlessness, value alienation, self-alienation and social isolation. The concept of social isolation refers to the loss of close contact with others, the collective and society. From the perspective of the external environment, it explained the impact of alienation from the environment on individuals. Work alienation is not limited to individual psychological analysis but is also closely related to the community location or governance environment. Regarding the core concept of self-separation, Franks and Marolla^25^ further divided alienation into three aspects: “despised self”, “disguised self” and “separated self”. Karasek^26^ pointed out that the "separated self" could best represent the core of work alienation, i.e., such separation within the work context, and further suggested that this separation may be caused by a lack of intrinsic rewards for work or by a lack of support and cooperation from the external environment^27^.

“Work area” is a concept in organizational management and refers to the division and setting of workplace according to its different functions and roles in an attempt to improve work efficiency and safety^28^. As work area is mainly used for the division of internal areas and arrangement of facilities, it focuses on a reasonable and coordinated relationship between work environment and human body structure, function and psychology from the perspective of physical characteristics and human engineering to render a workplace suitable for the demands of people’s physical and mental activities and improve work efficiency^29^. Apart from the division of work areas inside an organization, work area also involves the characteristics of the organization’s external environment that are present in the workplace^30^. The concept of work area is macroscopically classified, from the perspective of social characteristics, based on the factors in an employee’s workplace, such as its community environment, population distribution, or governance model. China has always been a country with a dichotomy between urban and rural areas; even within a city, there is a natural distinction between the centre (urban) and the suburbs (rural). This division is primarily a classification of geographical administrative division; moreover, this geographical classification is based on the social characteristics and population density of a focal region, as well as its grassroots community governance model. As community front-line workers, grassroots judicial administrators cannot achieve their tasks without community support. The separation from the community environment will lead to a rise in their work alienation. According to the social structure separation in China’s urban–rural dichotomy, the difference in the location of a community makes a significant difference in the mode of governance between the "stranger society" and the "acquaintance society"^31^. Different regions reflect different community environmental cultures and governance models and provide different community support. Compared with that in the suburbs, the community environment in the central districts is more complex, making the work more difficult and providing less support to workers in it^4^.

Previous studies on work alienation regarding both corporate and public employees have focused only on internal environment, such as organizational centralization^4,32^, interpersonal relationships within an organization^3^, and organizational red tape^33^. Less attention has been given to research on the work alienation caused by the external environment of the organization based upon organizational location. Additionally, most of the current research on work alienation is oriented towards psychological explanations while ignoring sociological explanations of the external community environment. Within China's dualistic urban–rural structure of society, this study has classified work area into two types from the perspective of the social feature of the external environment of the grassroots judicial offices: central districts (urban) and suburbs (rural). Since grassroots judicial administrators work in different areas, they will receive different community support under different community governance models, which will have an impact on the degree of their work alienation. Thus, Hypothesis 1 is as follows:

#### ***H1:***

*The work area affects the work alienation of grassroots judicial administrators, in which workers in the central districts have higher work alienation than workers in the suburbs.*

*Other influencing factors of work alienation* Employees’ work alienation is affected by many factors. Mottaz^11^ pointed out that the working environment has a significant effect on work alienation, and it is the most important of the many influencing factors. Indeed, its influence is stronger than that of employee background factors^34^. Kohn and Schooler^35^ concluded that work alienation includes both external and internal alienation: external alienation includes a lack of precise supervision, objective work factors, etc., and internal alienation includes work complexity, monotonicity, etc. In the same organization, different work contents determine the complexity and monotony of work to a certain extent. Especially in a highly hierarchical organization, work content that focuses only on complicated rules and procedures will greatly strengthen the sense of psychological powerlessness and meaninglessness^33^. Grassroots judicial administration includes internal affairs management, comprehensive social governance, criminal correction and people’s mediation. Internal work mainly refers to internal affairs management in the judicial office and the completion of the relevant administrative work of maintaining social stability assigned by the judicial administrative organs at higher levels and the township people’s government (sub-district offices), such as form summary, material sorting, or law enforcement inspection. Such work content is trivial and complex, repetitive and monotonous. Comprehensive social governance refers to the organization of publicity and education on the rule of law and the provision of grassroots legal services. People’s mediation mainly refers to assisting local governments in resolving and mediating disputes among community residents. Criminal correction refers to the implementation of community correction, resettlement, help and education of former prisoners. The latter three types of work content involve more contact with community residents, where grassroots judicial administrative workers have more leeway and freedom to decide working form and working method. If a staff member is responsible primarily for administration or the internal affairs of the judicial office, he or she must master complicated work procedures, and there is little room for him independent decisions, thereby increasing work alienation.

***H2:*** *Work content affects work alienation: workers engaged primarily in routine work have higher work alienation than professional workers. That is, grassroots judicial administrators who are in charge of internal work have higher work alienation than other workers.*

Position level can also affect employees’ work alienation. The higher their administrative level is, the more opportunities there are for employees to participate in decision-making and make independent decisions^36^ . Amid the same degree of organizational power centralization, the higher an individual’s position is, the more work autonomy he or she has, thereby reducing work his or her alienation^4,32^.

#### ***H3:***

*Position level affects work alienation: the higher the administrative level, the lower the work alienation.*

In addition to work environment and work characteristics, numerous studies have shown that the personal background of employees also has an impact on work alienation. Gender and age have an effect on employees’ work alienation. As age increases, women’s work alienation increases, while men’s work alienation decreases^37^. Employees with a high level of education and professional mismatch tend to have higher work alienation^9^, and the fit between employees’ professional education background satisfies the job requirements and affects work alienation^14^. In the case of grassroots judicial administrators, their work involves many basic legal tasks, such as law popularization. Those with a legal professional background are more competent in these work tasks, and they have lower work alienation than those with a nonlegal professional background. Additionally, overqualification increases employees’ work alienation and leads to emotional exhaustion^38^. Grassroots judicial administrative work is complicated and repetitive, and the greater the number of years is, the more likely professional burnout and excessive competence are, which in turn enhances work alienation. In this study, the sociodemographic factors (age, gender, education, professional background, and working years) of grassroots justice administrators are used as control variables, and the effects of work environment and work characteristics on work alienation are examined carefully.

In summary, previous research rarely involves social management workers. Most studies on work alienation focus on manual and technical workers^24,39^, such as fast food workers^9^, truck drivers^40^, industrial workers^41^, airline staff^5^, pharmacy salespersons^42^, midwives^43^, IT technicians ^3^ or university faculty^6^. Few studies, however, have been conducted on work alienation among grassroots judicial administrators. Such studies have mostly explored the influencing factors on work alienation via employees' personal characteristics and internal organization environment, neglecting the impact of the external environment of an organization’s location. Grassroots judicial administrators directly participate in community governance, social services and administrative law enforcement. Therefore, exploring the relationship between work area and work alienation can theoretically provide a new perspective for observing and explaining the work situation of grassroots judicial administrators while practically enabling organizations to respond to work alienation and apply positive coping strategies for it, which is the main purpose of this study.

## Methods

*Study design* This study is an empirical analysis of the work alienation of grassroots judicial administrators. Based on the literature review, considering the research team’s relationship network and the availability of research objects, all grassroots judicial administrators in Guangzhouwere taken as the sampling frame, and stratified random sampling and structured questionnaires were used to conduct a cross-sectional quantitative study.

The object of this study is grassroots judicial administrators. It should be noted that the factor of internal organizational environment characteristics is not covered in this study. The focal grassroots judicial offices are positioned within a highly hierarchical system with extremely high homogeneity in terms of organizational structure, internal working environment and work norms, and there are basically no differences across offices. Meanwhile, the relationship between coworkers and work atmosphere inside a judicial office may impact work alienation, as each judicial office usually has 5 employees who are very close to each other. The relationship between coworkers and work atmosphere reflects the hierarchy in their judicial office more than the circumstance of individual judicial administrative workers. Moreover, the questionnaire does not interrogate the relationship between coworkers and work atmosphere inside their judicial office to reduce the sensitivity of test questions and improve the probability of successful interviews. Therefore, their physical environment and coworkers’ relations within their grassroots judicial offices were excluded. This study also focuses on the Guangzhou area, so all the judicial administrators are in the same sociocultural context. This study is exploratory in nature, focusing on exploring the relationship between the work area, work characteristics (main work responsibilities and administrative post level) of grassroots judicial administrators and their work alienation.

*Setting and participants* Guangzhouis at the forefront of China’s reform and opening-up; it is a superlarge city and a pilot area for China’s judicial administrative reform. Compared with the typical absence of grassroots judicial offices at the village and township level in rural areas of other cities, the urban and rural areas in the jurisdiction of Guangzhou are fully covered by grassroots judicial offices; thus, a complete picture of the focal grassroots judicial administrative staff can be more effectively illustrated through better representativeness. The exploration of the work alienation of the judicial administrators in Guangzhoucan provide corresponding guidance for the country. Taking all grassroots judicial administrators in Guangzhouas the sample frame, the sample survey was carried out in Guangzhoufrom June to August 2019.

The grassroots judicial administrators selected in this study must meet all the following requirements: 1. They must be formal civil servants, excluding government employees who are not civil servants. 2. Their personnel appointment must be in the judicial bureau of a district in Guangzhou, with civil servants whose personnel appointment is in another government department or who are seconded to or temporarily working in a judicial office being excluded. 3. They must currently work in a grassroots judicial office. Judicial administrators currently working in bureaus (including the Guangzhou Judicial Bureau and the district judicial bureaus) are not grassroots personnel and are excluded from this study.

The stratified systematic sampling method was used (Supplementary Table S1, online, offers a more detailed description). The number of judicial administrative personnel varies according to the total population of the subdistrict or town where each judicial office is located, generally between 3 and 8. In this survey, if the number of personnel in a judicial office was ≤ 5, 2 were selected; if it was > 5, 3 were selected. First, based on the number of judicial offices in each district^44^, a certain number of judicial offices were randomly selected in each district. Second, 2–3 grassroots judicial administrators were randomly selected from each selected judicial office according to their internal serial numbers in the office. Third, the investigator conducted a questionnaire survey with the selected grassroots judicial administrators in the judicial office.

The questionnaire survey covered 11 districts and 132 judicial offices in Guangzhou. A total of 300 questionnaires were distributed, and 297 were retrieved, of which 288 were valid; thus, the valid questionnaire response rate was 96%. These sample data cover the judicial offices of all district-level administrative units in Guangzhou, with good representativeness and universality.

### Ethical considerations

This study involving human participants was reviewed and approved by the Ethics Committee of the School of Public Administration, Guangzhou University (Approval No. 2019-PBR021) and procedures were conducted in accordance with the Declaration of Helsinki. The relevant ethics protocols were strictly observed throughout the questionnaire survey process. Before the commencement of the questionnaire survey, the investigator briefly introduced the purpose of the survey, the principle of anonymity and voluntariness. All respondents provided written informed consent to participate in this study. If a grassroots judicial administrator refused to accept the survey, he or she would be replaced by the administrator with the next internal serial number to the right. The questionnaire was completed by the respondent or was completed via question-and-answer. The questionnaire was completed in a relatively independent environment, with the investigator providing timely on-site answers to interviewees’ questions. After the questionnaire was completed, the investigator immediately retrieved it to ensure the confidentiality of each respondent’s information.

### Variables

Work Alienation Scale (dependent variable). Work alienation in this study refers to the self-separation caused by work, and it also refers to the degree to which work does not provide inner satisfaction. The selection of the Work Alienation Scale was based primarily on the following factors: (1) it is recognized in academic circles and widely used, with good reliability and validity; (2) it has been tested in the Chinese context and translated into Chinese. The scale refers to the Work Alienation Scale prepared by Seeman^8^, which is based on Marx’s theory of alienation in the subjective sense and measures the “separated self”, focusing on employees’ separation within the scope of work. The Seeman scale has been widely used in cross-country work alienation research and comparisons and has become one of the classic paradigms for the development of the work alienation measurement scale^45^. Yang et al.^21^ and Robinson et al.^22^ translated the Work Alienation Scale into Chinese. This scale is a one-dimensional scale measuring inner work alienation. The scale includes a total of 7 questions, which measure whether their work is regarded by the employees as highly intensive, challenging, creative, focused, worthy of persistence, autonomous or boring. The scale adopts a dichotomous (Yes or No) response option, and each question is scored as 0 or 1. After reverse conversion of some items, the total score of the scale is between 0 and 7. The higher the score is, the stronger the work alienation. Based on the data from 288 grassroots judicial administrators in Guangzhou, the 7 scale items were used for item-analysis, and all passed. The KR-20 coefficient of internal consistency reliability of the scale was 0.537. Although the internal consistency reliability of the scale did not reach 0.7, the scale reliability was 0.51 and 0.50 in US and French samples, respectively^46^, so the reliability of the scale is acceptable. There was a strong negative correlation between the Work Alienation Scale and the Work Engagement Scale^47^ (Pearson correlation coefficient *r* = -0.574, *p* < 0.001), so the scale has good discrimination validity.

Work Area (independent *variable).* The work area is a dichotomous variable divided into “0” = “suburbs” and “1” = “central district” according to the location of the administrative area in which the judicial office is located in Guangzhou. According to Guangzhou’s 2019 urban administrative division map^48^ and the 2020 Guangzhou Statistics Yearbook^49^, the districts of Huadu, Zengcheng, Nansha, Conghua and Baiyun are the suburbs, and the six districts of Haizhu, Huangpu, Panyu, Yuexiu, Tianhe and Liwan are the central districts. The suburbs have a much lower population density than the central districts. After calculation, the population density of permanent residents is 6,735.04/km^2^ in the six central districts and 1,073.53/km^2^ in the five suburban districts (Supplementary Table S2, online, offers a more detailed description). In addition, in terms of the community governance model, the suburbs are mainly dominated by village committee autonomy, while the central district mainly depends on community committees. These districts are significantly different from the central districts in terms of population density and governance mode.

Work Characteristics (independent variables*)* The main work responsibilities and administrative post level were included. According to the judicial office’s responsibilities, grassroots judicial administrators have nine major tasks. Based on the nature of the work and the similarity of the content, this study categorizes work content into four categories: “1” = “internal work” (comprehensive work in the judicial office + internal affairs management), “2” = “comprehensive social governance” (administrative law enforcement + social security governance + legal services + law promulgation), “3” = “criminal correction” (community corrections + aftercare), and “4” = “people’s mediation”. Respondents may be responsible for multiple types of work tasks. This study asks about and records the type of work tasks that respondents spend the most time and energy on. According to the provisions of the *National Civil Service Law* and the administrative unit level of subdistrict and town judicial offices in Guangzhou, the administrative post levels can be divided into four levels from low to high: “1” = “clerk”, “2” = “officer”, “3” = “associate chief officer”, and “4” = “chief officer and above”.

Sociodemographic Variables (control variables*)* The gender, age, education level, professional background and working years of grassroots judicial administrators were included. Gender is a dichotomous variable: “0” = “male” and “1” = “female”. Age is the full year of age of the respondent at the time of the questionnaire survey. Education level is a dichotomous variable: “0” = “junior college education and below”, and “1” = “undergraduate education and above”. Professional background was originally a fill-in-the-blank question, and the respondents indicated their majors at different educational stages (junior college education and above) in turn. If a respondent’s major was law at any stage of education, professional background is coded “0” (major of law); otherwise, it is coded “1” (any major other than law). Working years refers only to the number of years the respondent has been engaged in grassroots judicial administration, that is, the respondent’s work time in the judicial office was counted; the respondent’s time spent working in the judicial bureau and on other types of work was excluded.

*Sampling information* A total of 288 grassroots judicial administrators were interviewed, of which 203 were males, accounting for 70.5%, and 85 were females, accounting for 29.5% (see Table 1). The age range was 20 to 57 years old, with an average age of 38.0 years (*SD* = 9.317). Among the grassroots judicial administrators, 206 had received an undergraduate education or above, accounting for 71.5%, and 82 had received a junior college education or below (including 6 with a high school education level), accounting for 28.5%. In terms of professional background, 199 graduates majored in law, accounting for 69.1%, and 89 graduates did not major in law, accounting for 30.9%. The graduates not majoring in law majored primarily in economics, administrative management and humanities and social sciences. The number of years worked in the grassroots judicial administration was between 1 and 33 years, and the average working time was 9.33 years (*SD* = 8.580); this was strongly positively correlated with age (*r* = 0.618, *p* < 0.001), showing that the number of years grassroots judicial administrators have worked in grassroots administration increases with age and that the team is stable, with relatively low overall mobility. The proportion of grassroots judicial administrators whose daily workplaces are in the central districts was close to 60%, and the workplaces of the remaining 40% are in the suburbs. Criminal correction (44.8%) work was the main daily work of the grassroots judicial administrators, followed by people’s mediation and internal work in the judicial offices, accounting for 28.1 and 15.6%, respectively. In terms of administrative post distribution, there were 81 chief officers and above, accounting for 28.1%; 50 associate chief officers, accounting for 17.4%; 87 officers, accounting for 30.2%; and 70 grassroots clerks, accounting for 24.3%. In summary, most grassroots judicial administrators are men; they are generally middle aged; their education level is relatively high; nearly three-quarters have received undergraduate education and almost all of them have received higher education; and most studied law or economics, management, humanities and social sciences, etc. Currently, the personnel pool is stable and has a reasonable distribution in terms of administrative levels and rich experience in judicial administration. The total number of personnel in the central districts is slightly higher than that in the suburbs.

**Table 1:** Status quo of the grassroots judicial administrators.

Variable	Mean^a^	SD	Min	Max
Gender (female)^b^	0.295	0.457	0	1
Age	38.049	9.317	20	57
Education level (undergraduate education and above)	0.715	0.452	0	1
Major (other than law)	0.309	0.463	0	1
Working years	9.330	8.580	1	33
Work area (central district)	0.597	0.491	0	1
Work responsibilities^c^				
Internal work	0.156	0.364	0	1
Comprehensive social governance	0.115	0.319	0	1
Criminal correction	0.448	0.498	0	1
People’s mediation	0.281	0.450	0	1
Administrative level^c^				
Clerk	0.243	0.430	0	1
Officer	0.302	0.460	0	1
Associate chief officer	0.174	0.379	0	1
Chief officer and above	0.281	0.450	0	1

a. The mean of dichotomous variable indicates the percentage of category assigned with higher value, such as “1” = “female” and “0” = “male”, and the mean of gender = . 295, indicating that women account for 29.5%; b. the category in parentheses is assigned “1”; c. the work responsibilities and administrative levels have undergone dummy variable conversions from four-choice category variables to four dichotomous variables with values of 0–1.

*Statistical methods* Statistical analysis was performed using SPSS 24.0 software. First, descriptive statistics (mean, standard deviation, etc.) were used to reveal the status quo of grassroots judicial administrators’ work alienation. Second, variables, such as gender and age, in relation to work alienation were separately analysed in terms of variance or correlation to explore the relationship between each variable and work alienation separately. Third, work alienation was taken as the dependent variable, work characteristics as the first-level independent variables, and work area as the second-level independent variables. Next, ordinary least squares linear regression analysis was conducted to further explore the factors significantly associated with work alienation while controlling for sociodemographic factors.

Since the data of this research were derived from a questionnaire sampling survey, regression analysis was finally conducted for all variables, Common Method Variance (CMV) may lead to systematic measurement errors and further skew the estimation of the true relationship between variables^50^. Therefore, the existence of CMV is tested for prior to official data analysis. Harman’s one factor method is used to include all factors in this paper into factor analysis, where 1 common factor is set to be extracted (without sub-extraction). The analysis shows that the explained variation caused by 1 factor is 16.987%; thus CMV does not exist in the data collected in this research, with 50%^51^ as the judgement criterion.

## Results

*Status quo of work alienation* After statistical analysis of items and total scores of work alienation, as Table 2 shows, the problems of high work intensity (66.7%) and lack of autonomy (75.7%) among the grassroots judicial administrators were the most prominent, with more than two-thirds of the respondents reporting these problems. In comparison, the problems of “lack of challenge” (17.7%) and “boring” (12.8%) were not prominent. The total mean of work alienation = 2.50, and the standard deviation = 1.512. The overall work alienation of the grassroots judicial administrators was thus at a moderately low level. With work alienation as the dependent variable, before the regression analysis, a histogram was first drawn to test its normality. Skewness = 0.737, kurtosis = 0.655, the Kolmogorov–Smirnov test passed (*p* < 0.001), and the normality of the data distribution was acceptable.

**Table 2:** Work alienation.

Item	Mean	SD	Min	Max
High work intensity	0.667	0.472	0	1
Lack of challenge	0.177	0.382	0	1
Lack of creativity	0.288	0.454	0	1
Unable to focus	0.292	0.455	0	1
Not worth persisting	0.191	0.394	0	1
Lack of autonomy	0.757	0.430	0	1
Boring	0.128	0.335	0	1
Total score	2.500	1.512	0	7

The independent variables in relation to work alienation were separately analysed in terms of variance or correlation. In Table 3, female grassroots judicial administrators (2.56) had slightly higher work alienation than men (2.47), but there was no significant difference (*p* = 0.639). For those with different professional backgrounds, work responsibilities and administrative levels, there was no significant difference in their work alienation. Education level and work area were significantly related to work alienation: those with a junior college education and below had significantly higher work alienation (2.98) than those with an undergraduate education and above (2.31), *t* = 3.431, *p* < 0.01; those working in the central districts had significantly higher work alienation (2.89) than those working in the suburbs (1.92), *t* = −5.599, *p* < 0.001. Age and years worked had a significant positive correlation with work alienation, and the Pearson correlation coefficients were 0.131 and 0.135, respectively (*p* < 0.05), indicating that the older the age and the greater the number of years worked, the stronger the work alienation was.

**Table 3:** One-way variance/correlation analysis of work alienation.

Variable	Category	Work alienation			
		*Mean*	*SD *^*a*^	Statistic	*p*
Gender	Male	2.47	1.581	*t* = −0.469	0.639
	Female	2.56	1.340		
Education level	Junior college education and below	2.98	1.832	*t* = 3.431	0.003**
	Undergraduate education and above	2.31	1.322		
Professional background	Law	2.54	1.588	*t* = 0.632	0.528
	Other than law	2.42	1.330		
Work area	Suburbs	1.92	1.224	*t* = -5.599	< 0.001***
	Central districts	2.89	1.565		
Work responsibilities	Internal work	2.53	1.455	*f* = 1.856	0.137
	Comprehensive social governance	3.03	1.960		
	Criminal correction	2.34	1.428		
	People’s mediation	2.52	1.441		
Administrative level	Clerk	2.37	1.590	*f* = 0.424	0.736
	Officer	2.53	1.453		
	Associate chief officer	2.68	1.684		
	Chief officer and above	2.47	1.406		
Age				*r* = 0.131	0.026*
Working years				*r* = 0.135	0.022*

* *p* < 0.05, ** p < 0.01, *** *p* ≤ 0.001; a. SD = standard deviation.

*Hierarchical regression analysis* With work alienation as the dependent variable and gender, age, education level, professional background and working years as control variables, a regression analysis was performed. From the analysis results of Model 1 in Table 4, it can be seen that when controlling for other variables, gender and professional background had no significant effect on work alienation, and the effect of age on work alienation became negligible. Education level had a significant effect on work alienation, as well as the number of working years. The nonstandard regression coefficient *B* = −0.662, *p* = 0.001, indicating that the work alienation of the grassroots judicial administrators with an undergraduate education or above was lower by 0.662 points on average than that of those with a junior college education or below. Taking the work characteristics factors as the first-level independent variables and including them in the regression analysis, Model 2 shows that after the inclusion of work characteristics, education level still had a significant effect on work alienation, *B* = −0.627, *p* < 0.01; gender, age and professional background still had no significant correlation with work alienation. Taking the internal work of the judicial office as a reference, there was no correlation between work responsibilities and work alienation. In the same way, administrative level also had no effect on work alienation. Finally, work area as the second-level independent variable, enters into regression Model 3. The work area of the grassroots judicial administrators had a significant effect on their work alienation, *B* = 0.917, *p* < 0.001, with the work alienation of the personnel working in the central districts being higher by 0.917 points on average than that of those working in the suburbs. It can be seen from the standardized regression coefficients of the independent variables in Model 3 that the work area *Beta* = 0.298 and the education level *Beta* = −0.151 in the regression model, so work area had a greater effect on work alienation than education level.

**Table 4:** Regression model of work alienation.

Variable	Model 1			Model 2			Model 3		
	*B *^*a*^		*Beta*	*B*		*Beta*	*B*		*Beta*
(Constant)	2.392	(0.454)		2.358	(0.584)		2.304	(0.560)	
Gender (female)	0.162	(0.196)	0.049	0.181	(0.197)	0.055	0.059	(0.191)	0.018
Age	0.015	(0.012)	0.090	0.020	(0.016)	0.121	0.003	(0.016)	0.016
Education level (undergraduate education and above)	−0.662**	(0.202)	−0.198	−0.627**	(0.228)	−0.188	−0.505*	(0.220)	−0.151
Professional background (other than law)	−0.252	(0.201)	−0.077	-0.328	(0.208)	−0.101	−0.149	(0.202)	−0.046
Working years	0.006	(0.014)	0.035	0.005	(0.014)	0.026	0.016	(0.014)	0.092
Work responsibilities (internal work as the reference)									
Comprehensive social governance				0.447	(0.349)	0.094	0.266	(0.337)	0.056
Criminal correction				−0.146	(0.307)	0.048	−0.227	(0.295)	−0.075
People’s mediation				−0.145	(0.290)	−0.043	−0.315	(0.280)	−0.094
Administrative level (clerk as the reference)									
Officer				0.002	(0.291)	0.001	0.036	(0.279)	0.011
Associate chief officer				−0.171	(0.376)	−0.043	−0.005	(0.361)	−0.001
Chief officer and above				−0.240	(0.408)	−0.071	−0.216	(0.391)	−0.064
Work area (central district)							0.917***	(0.182)	0.298
R-squared	0.060			0.077			0.155		

**Note:** * *p* < 0.05; ** *p* < 0.01; *** *p* ≤ 0.001; a. standard error in parentheses.

**Figure 1:**
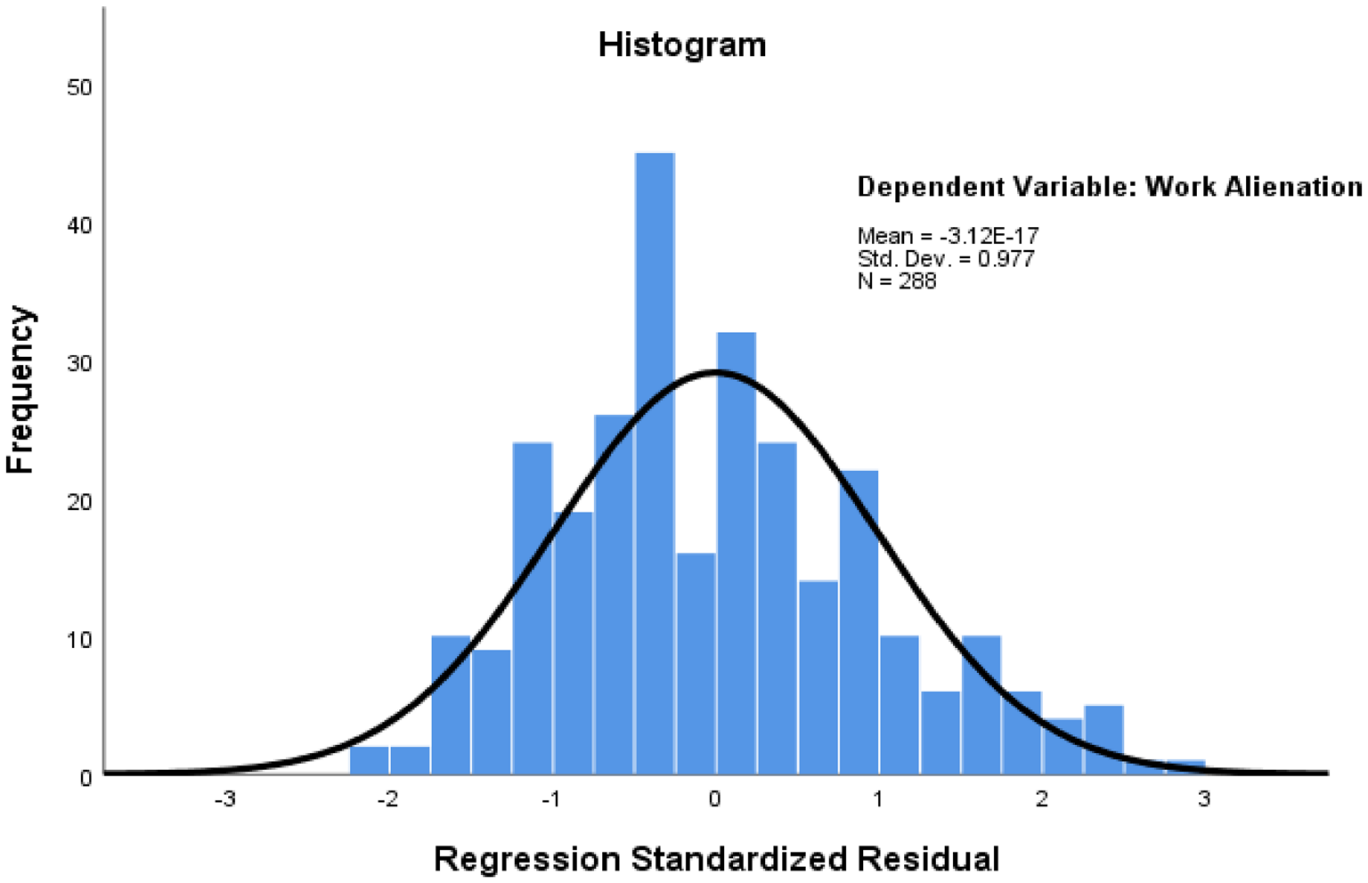
Regression standardized residual histogram.

**Figure 2:**
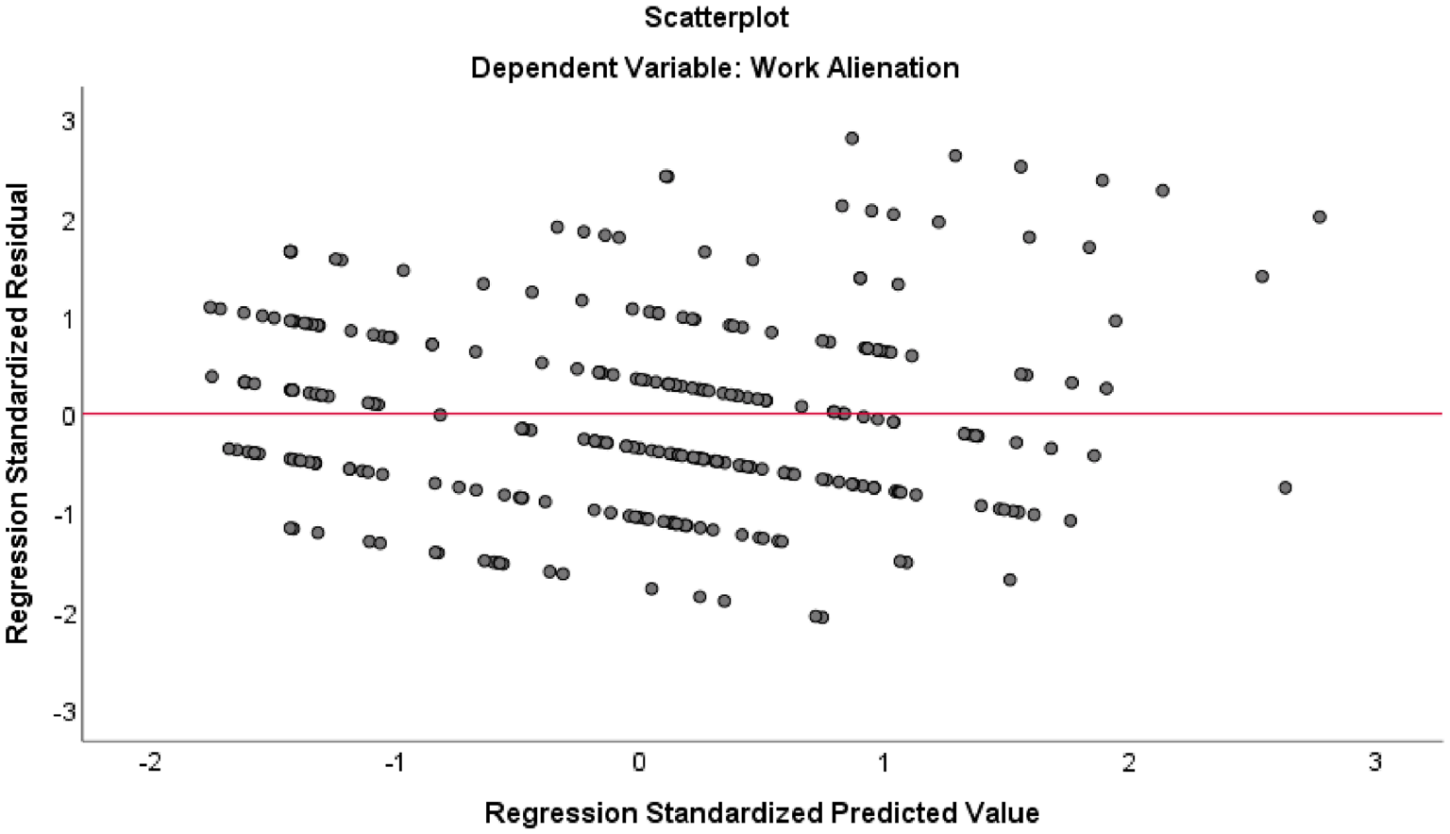
Regression standardized predicted value and residual scatter plot.

The applicable conditions of the Model 3 regression model were tested. The Durbin-Watson test value = 0.252, which is close to 2, indicating a very small possibility of autocorrelation among model residuals and that the observation data of the model can be regarded as independent of each other. The model standardized residual histogram in Fig. 1 indicates that the model residuals are subject to a normal distribution (Kolmogorov–Smirnov test *p* < 0.001). In the residual scatter plot in Fig. 2, the model residuals do not change with the size of the model predicted values or the values of any of the variables, and the residuals are randomly distributed above and below the “0” reference line, with homogeneity of variance. The casewise diagnostics method was used to identify strong influential points (standardized residuals greater than 3), with none being found in the model. The multicollinearity of the independent variables in Model 3 was further tested, and the variance inflation factor (VIF) values of the independent variables were between 1.082 and 4.410, and all were less than 5, indicating no serious collinearity among the independent variables^52^. Finally, the explanatory power of the models was tested. Model 1 included only the sociodemographic factors as control variables, and the coefficient of determination *R-squared* = 0.060 (*adjusted R-squared* = 0.043); after the inclusion of the work characteristics factors, the Model 2 *R-squared* = 0.077 (*adjusted R-squared* = 0.060), a slight increase of 0.017 (*F* change = 0.826, *p* = 0.551) compared to the value in Model 1. For Model 3. *R-squared* = 0.155, a significant increase of 0.078 compared with Model 2 (*F* change = 25.401, *p* < 0.001). This indicates that when the work area variable is added to the regression model, the overall explanatory power of the models is significantly improved, and the work area of the grassroots judicial administrators has stronger explanatory power than the other factors.

## Discussion

This study found that the grassroots judicial administrative personnel pool has a relatively reasonable overall structure and high professional quality and is relatively stable. The overall work alienation of the grassroots judicial administrators is at a moderately low level, but problems with high work intensity and lack of autonomy are prominent. With work alienation as the dependent variable and with only control variables included in the regression model, only education level is significantly associated with work alienation. When the work characteristics factors – work responsibilities and administrative level – are further included in the regression model, the explanatory power of the regression model is minor and insignificantly improved, and the influence of education level is still significant. Finally, work area is included and it has a greater influence on work alienation with the control of other variables. For grassroots judicial administrators, the lower their education level is, the stronger the work alienation; only education level can play a controlling role in the final model. Those working in the central districts have higher work alienation than those working in the suburbs (H1 supported). Work responsibilities (H2 rejected) and administrative post level (H3 rejected) are not significantly associated with work alienation.

Studies have pointed out that there are differences in the level of work alienation among different types of labour groups (Zhou & Long^11^), and such differences are also reflected in the fact that the same factors have different effects on work alienation among different types of labour groups. There is a significant correlation between education level and the work alienation of grassroots judicial administrators. Although education level is entered into the model as a control variable, it still provides some useful insights. In this study, the higher the education level was, the lower the work alienation of the grassroots judicial administrators, and vice versa. DiPietro and Pizam^9^ conducted a study on work alienation among employees and managers in the American fast food industry and found that education level has a significant effect on work alienation, but there is a positive correlation between the two: the higher the education level is, the higher the work alienation. To understand these two completely opposite research findings, it is necessary to explore and explain the work ability needs of different types of labour groups^53^. For manual labour groups, such as fast food workers and truck drivers, there are no requirements to have a high level of education, and employees with higher education levels cannot express their knowledge value through their work and are more likely to have a sense of powerlessness and value alienation. Thus, their work alienation is positively correlated with their education level. Grassroots judicial administrators need to manage the intricate legal affairs of local communities, carry out highly professional criminal correction work, and complete a series of judicial administrative tasks. They need a wealth of professional knowledge in law, sociology, and psychology. A high education level makes it easier for them to execute grassroots judicial administrative work, thereby enhancing their sense of competence and recognition of the value of their own work and reducing their work alienation.

The area where the judicial office is located has a significant effect on the work alienation of grassroots judicial administrators. The work alienation of the judicial administrators working in the suburbs is significantly lower than that of those working in the central districts. According to the investigation, the reasons are as follows. The first issue is the complexity of the work. The density of permanent residents in the central districts is much higher than that in the suburbs (Supplementary Table S2, online, offers a more detailed description). In regard to the judicial administrative work of a certain community in an area, high population density indicates that population service management and legal affairs in the community are more complex and that more grassroots judicial administrative work needs to be carried out^54^. Thus, the work pressure and work intensity are naturally higher than in areas with lower population densities. The second issue is the differences in social governance models between suburban and central districts. Regarding the social management work and legal services directly performed by grassroots judicial administrators, many tasks need to be carried out in cooperation with grassroots political organizations in the area. In the central districts, community governance is mainly carried out through the subdistrict office and residents’ committee; in the suburbs, it is mainly carried out through the town government and villagers’ committee. The village is an acquaintance society, and each village has established local rules that have strong binding and controlling power over the villagers^55^. On the other hand, a subdistrict is often divided into communities of different numbers, with high heterogeneity among community members. These communities are societies of strangers. For historical and social reasons unique to China, villagers’ committees and villagers’ autonomy have a longer history and a more stable form, while residents’ committees are experiencing difficulty achieving autonomy at present^56^. Grassroots judicial administrators in the suburbs can more easily secure villagers’ autonomous and conscious cooperation when carrying out judicial administration, and the work pressure in the suburbs is lighter. In the central district governance model, however, the community composition is more complicated, and community members are not connected by family ties but are atomized individuals who gather from different places to make a living and do not know each other. Grassroots judicial administrators experience less cooperation from residents in the central districts, and it is difficult to carry out their work, so their work pressure and work alienation increase accordingly. Although Fedi et al.^36^ pointed out that the different positions of employees in the organization have different effects on their work alienation, in the civil service system of the grassroots judicial administration, the administrative level and work responsibilities are not related to work alienation. For grassroots judicial administrators, the external environmental characteristics of their work are more significantly related to work alienation than to internal differences.

## Conclusion

As an exploratory study, this study adopts a cross-sectional method, which is its main limitation. In addition, this study measured only the relationship between the objective external work environment and work alienation; it did not address the relevant subjective psychological influencing factors, such as self-esteem^57^ or sense of achievement^58^. Further studies can consistently examine the effect of the external environment after considering subjective factors. For grassroots judicial administrators, high work intensity and lack of autonomy at work are the most prominent manifestations of their work alienation. To reduce work alienation among grassroots judicial administrators, specific follow-up training and continuing education should be provided to improve their professional knowledge level. For those in the central districts, it is beneficial to strengthen the contact and interaction between the grassroots judicial offices and the local government organizations and community residents and promote the self-organization ability of communities in the central districts to improve the overall quality of grassroots judicial administrative work and reduce work alienation.

## Supplementary Information

Supplementary Information.

## Data Availability

The datasets generated and analysed in the present study are available in the figshare repository, https://doi.org/10.6084/m9.figshare.19632588.
