# Supplementary material for: The effect of work area on work alienation among China’s grassroots judicial administrators
Source: Sci Rep. 2022 Nov 5;12:18784. doi: 10.1038/s41598-022-23526-w (PMC9637184; doi:10.1038/s41598-022-23526-w)
Supplement: Supplementary file 1 — Supplementary Information. [file 41598_2022_23526_MOESM1_ESM.docx]

Supplementary Information to manuscript:

**The Effect of Work Area on Work Alienation among China’s Grassroots Judicial Administrators**

Nian Liu^1^, Meiling Zhang^2^, Boya Feng^3,4^

^1^Department of Sociology, Guangzhou University, Guangzhou, 510006, China

^2^Social Construction Committee of Guangdong Provincial People's Congress, Guangzhou, 510062, China

^3^The Department of Social Work，Tunghai University, Taichung, 407224, Taiwan

^4^School of Law, Guangdong University of Technology, Guangzhou, 510520, China

Corresponding authors:

Nian Liu, liunian@gzhu.edu.cn

Boya Feng, fengbora@hotmail.com

| District | Number of judicial offices in the district | Number of judicial offices surveyed | Percentage of judicial offices surveyed (%) | Number of respondents | Percentage of respondents (%) |
| --- | --- | --- | --- | --- | --- |
| Baiyun | 18 | 18 | 13.6 | 52 | 18.1 |
| Huangpu | 15 | 15 | 11.4 | 35 | 12.2 |
| Panyu | 16 | 13 | 9.8 | 33 | 11.5 |
| Tianhe | 21 | 15 | 11.4 | 28 | 9.7 |
| Yuexiu | 18 | 15 | 11.4 | 27 | 9.4 |
| Haizhu | 18 | 13 | 9.8 | 27 | 9.4 |
| Huadu | 8 | 8 | 6.1 | 23 | 8.0 |
| Liwan | 22 | 14 | 10.6 | 22 | 7.6 |
| Zengcheng | 11 | 10 | 7.6 | 22 | 7.6 |
| Conghua | 8 | 7 | 5.3 | 13 | 4.5 |
| Nansha | 8 | 4 | 3.0 | 6 | 2.1 |
| Total | 163 | 132 | 100.0 | 288 | 100.0 |

**Table S1.** Sampling distribution

| Work Area | District | Resident Population (10,000) | Population Density (persons/km^2^) | Number of Community Committees | Number of Village Committees |
| --- | --- | --- | --- | --- | --- |
| Suburb | Conghua | 64.95 | 329 | 44 | 221 |
|  | Zengcheng | 126.01 | 780 | 37 | 282 |
|  | Nansha | 79.61 | 1,016 | 19 | 58 |
|  | Huadu | 110.72 | 1,141 | 50 | 188 |
|  | Baiyun | 277.96 | 3,439 | 247 | 118 |
| Central | Panyu | 182.78 | 3,449 | 247 | 92 |
|  | Huangpu | 115.12 | 5,035 | 99 | 28 |
|  | Liwan | 101.2 | 17,124 | 193 | 0 |
|  | Tianhe | 178.85 | 18,566 | 200 | 0 |
|  | Haizhu | 172.42 | 19,073 | 257 | 0 |
|  | Yuexiu | 120.97 | 35,790 | 267 | 0 |

**Table S2.** Statistics on population density and grassroots self-government organizations in Guangzhou by district
